# Editorial: Molecular mechanism of ageing and therapeutic advances through targeting glycative and oxidative stress

**DOI:** 10.3389/fphar.2023.1324446

**Published:** 2024-03-06

**Authors:** Jaba Tkemaladze

**Affiliations:** Free University of Tbilisi, Tbilisi, Georgia

**Keywords:** aging, stress, glycative stress, oxydative stress, senescence

The inexorable passage of time has always intrigued humanity, prompting an incessant quest for unravelling the mysteries underlying the ageing process. However one should distinguish between the ageing of the organism and cellular senescence (Tkemaladze, 2023). During the process of cellular ageing (senescence), cells lose the ability to divide and become senescent either due to reaching the Hayflick limit (Hayflick, 1965) or due to stress factors. The theories of cellular senescence can be broadly categorized into two groups. The first group suggests that ageing is a genetically programmed event, potentially involving specific “ageing genes” or the depletion of useable genetic information. The second group views senescence as the outcome of accumulating damage to cell structures or errors in information-containing molecules (Harman, 1968). One prevalent theory in this category is the “error catastrophe” hypothesis, which proposes that the gradual accumulation of random errors in proteins responsible for vital cellular functions leads to a threshold beyond which cell function ceases. These theories imply that cytoplasmic events may significantly contribute to the development of cellular senescence.

While the intricate mechanisms of ageing remain a conundrum, recent scientific strides have shed light on the pivotal roles played by glycative and oxidative stress in this complex biological phenomenon. The intertwined nature of these two processes, acting as catalysts in cellular deterioration, has emerged as a promising avenue for potential therapeutic interventions (Jiang et al.). As the understanding of these molecular intricacies deepens, the prospect of targeted interventions to decelerate the ageing process and mitigate age-related diseases has become increasingly plausible (Cai et al.; Ma et al.).

This editorial endeavours to delve into the fascinating world of the molecular mechanisms of ageing, emphasizing the significance of comprehending glycative and oxidative stress in this context. Additionally, it aims to underscore the recent breakthroughs in therapeutic strategies that harness these insights, potentially offering a glimmer of hope in the pursuit of extending human longevity and improving the quality of life in the elderly population. Рecent advancements in molecular biology and biotechnology have illuminated the critical roles of glycative and oxidative stress in mediating cellular ageing and the associated pathophysiological consequences (Xu et al.).

Glycation, a non-enzymatic reaction between reducing sugars and amino groups of proteins, lipids, and nucleic acids, culminates in the formation of advanced glycation end products (AGEs). These AGEs have been implicated in the disruption of cellular homeostasis, instigating a cascade of detrimental effects including oxidative stress, inflammation, and impaired cellular function. Concomitantly, oxidative stress, characterized by an imbalance between the production of reactive oxygen species (ROS) and the antioxidant defence system, induces cellular damage and accelerates the ageing process. The intricate interplay between glycative and oxidative stress contributes to the deterioration of cellular components, ultimately manifesting as age-related pathologies such as neurodegenerative diseases, cardiovascular ailments, and metabolic disorders.

Oxidative stress, a fundamental component in the process of ageing and the development of various age-related diseases is a condition characterized by an imbalance between the production of reactive oxygen species (ROS) and the ability of a biological system to readily detoxify these reactive intermediates or repair the resulting damage. ROS, including free radicals such as superoxide anion (O_2_-), hydroxyl radical (OH.), and non-radical species like hydrogen peroxide (H_2_O_2_), are generated during normal cellular metabolism and serve crucial roles in cell signalling and homeostasis. However, when produced excessively or not effectively neutralized by the antioxidant defence system, these ROS can inflict significant damage on cellular components, including lipids, proteins, and nucleic acids.

Cellular sources of ROS are diverse and include mitochondria, peroxisomes, endoplasmic reticulum, and cytoplasmic enzymes. Mitochondria, in particular, are one of the major sites of ROS production during the process of oxidative phosphorylation. The leakage of electrons from the electron transport chain can lead to the formation of superoxide radicals, which can subsequently give rise to other highly reactive ROS. Additionally, inflammatory cells, such as macrophages and neutrophils, produce ROS as a part of the immune response, aiding in the eradication of pathogens. However, uncontrolled and excessive ROS production from these immune cells can lead to tissue damage and chronic inflammation.

The detrimental effects of ROS on cellular components are numerous. Lipid peroxidation, initiated by the attack of ROS on polyunsaturated fatty acids in cellular membranes, can lead to the disruption of membrane integrity and function. Furthermore, ROS-induced modifications of proteins can impair their structure and function, leading to enzyme inactivation, altered signalling pathways, and the formation of protein aggregates. Damage to nucleic acids, particularly DNA, by ROS can result in mutations, and genomic instability, and ultimately contribute to cellular senescence or apoptosis.

In response to the threat posed by ROS, cells have evolved intricate antioxidant defence systems to counteract oxidative stress. These defence mechanisms include enzymatic antioxidants such as superoxide dismutase (SOD), catalase, and glutathione peroxidase, as well as non-enzymatic antioxidants like vitamins C and E, glutathione, and various phytochemicals. These antioxidants neutralize ROS directly or indirectly by scavenging free radicals, repairing oxidative damage, or regenerating other antioxidants. Disruption of this delicate balance between ROS production and the antioxidant defence system can result in a state of chronic oxidative stress, contributing to the ageing process and the development of various age-related pathologies, including neurodegenerative diseases, cardiovascular disorders, cancer, and metabolic dysfunctions.

Researchers and clinicians have increasingly recognized the significance of targeting oxidative stress as a therapeutic strategy to mitigate the effects of ageing and age-related diseases. This approach involves not only the development of novel antioxidants but also the exploration of lifestyle modifications, including dietary interventions and regular physical exercise, which can bolster the endogenous antioxidant defence system. Furthermore, understanding the intricate interplay between oxidative stress and other cellular processes, such as inflammation and cellular senescence, has provided insights into the development of integrated therapeutic approaches that target multiple pathways simultaneously, aiming to promote healthy ageing and extend the health span of individuals.
